# Genetic and epidemiological analysis of norovirus from children with gastroenteritis in Botswana, 2013–2015

**DOI:** 10.1186/s12879-018-3157-y

**Published:** 2018-05-30

**Authors:** Kgomotso Makhaola, Sikhulile Moyo, Kwana Lechiile, David M. Goldfarb, Lemme P. Kebaabetswe

**Affiliations:** 1 Department of Biological Sciences and Biotechnology, Botswana International University of Science and Technology, Private Bag, 16 Palapye, Botswana; 2 Botswana Harvard AIDS Institute Partnership, Gaborone, Botswana; 3 Harvard T.H Chan School of Public Health, Boston, MA USA; 4 Botswana-UPenn Partnership, Gaborone, Botswana; 5 Department of Pathology, University of British Columbia, Vancouver, Canada

**Keywords:** Norovirus, GII.4 variants, GII.Pe-GII.4, Botswana, Genotyping

## Abstract

**Background:**

Norovirus is a leading cause of viral gastroenteritis worldwide with a peak of disease seen in children. The epidemiological analysis regarding the virus strains in Africa is limited. The first report of norovirus in Botswana was in 2010 and currently, the prevalence and circulating genotypes of norovirus are unknown, as the country has no systems to report the norovirus cases. This study investigated the prevalence, patterns and molecular characteristics of norovirus infections among children ≤5 years of age admitted with acute gastroenteritis at four hospitals in Botswana.

**Methods:**

A total of 484 faecal samples were collected from children who were admitted with acute gastroenteritis during the rotavirus vaccine impact survey between July 2013 and December 2015. Norovirus was detected using real-time RT-PCR. Positive samples were genotyped using conventional RT-PCR followed by partial sequencing of the capsid and RdRp genes. Norovirus strains were determined by nucleotide sequence analysis using the online Norovirus Genotyping Tool Version 1.0, and confirmed using maximum likelihood tree construction as implemented in MEGA 6.0.

**Results:**

The prevalence of norovirus was 9.3% (95% CI 6.7–11.9). The genotype diversity was dominated by the GII.4 strain at 69.7%. This was followed by GII.2, GII.12 each at 9.1%, GI.9 at 6.6% and GII.6, GII.10 each at 3.0%. The most common combined RdRp/Capsid genotype was the GII.Pe/GII.4 Sydney 2012. Norovirus was detected during most part of the year; however, there was a preponderance of cases in the wet season (December to March).

**Conclusion:**

The study showed a possible decline of norovirus infections in the last 10 years since the first report. An upward trend seen between 2013 and 2015 may be attributable to the success of rotavirus vaccine introductions in 2012. Knowledge of circulating genotypes, seasonal trends and overall prevalence is critical for prevention programming and possible future vaccine design implications.

**Electronic supplementary material:**

The online version of this article (10.1186/s12879-018-3157-y) contains supplementary material, which is available to authorized users.

## Background

Globally, noroviruses are a leading cause of acute gastroenteritis [1]. Its symptoms include nausea, diarrhoea, vomiting, stomach cramps and fever [2]. While these symptoms can be self-limiting in healthy individuals, children, elderly and immune-compromised individuals often experience prolonged symptoms [3, 4]. The shedding of norovirus has also been shown in asymptomatic individuals [5], and its transmission occurs mainly by the faecal-oral route, through either contaminated food or water sources [6], person to person contact or through aerosols [7]. Additionally, norovirus has been shown to have low infectious dose [8]. Currently, there is no antiviral treatment for norovirus infections, and vaccines are in late development stages [9, 10]. Noroviruses are single stranded, small non-enveloped RNA viruses that belong to the Caliciviridae family [11, 12]. Their genome is approximately 7.5 kb long and comprises three to four open reading frames (ORF), [12, 13]. ORF-1 encodes a large polyprotein, which is cleaved by virus-encoded protease, into six non-structural proteins that include the RNA-dependent RNA polymerase (RdRp) [14]. ORF-2, ORF-3 and ORF-4 encode the major capsid protein, VP1, minor structural protein, VP2 and virulence factor, respectively. The ORF-4 is unique to the murine norovirus cluster [6, 8, 14]. When using complete amino acid sequences of the capsid protein, noroviruses cluster into seven genogroups [10, 15], and each genogroup is subdivided into several genotypes. While genogroups I, II and IV have been shown to infect humans [5, 8], GII.4 is the predominant genotype globally, and every few years new GII.4 variants surface that rapidly replace the dominant circulating strain [16]. Several factors have been suggested that enable this strain to dominate infections and these are: high mutation rates, wide receptor specificity, and short-term immunity compared to other strains [2, 6]. Routinely, the norovirus genotype determination is by either the partial capsid or RdRp gene sequences. However, noroviruses being RNA viruses undergo recombination at ORF1/ORF2 that leads to a single virus strain clustering under different genotypes when different genomic regions are used for sequencing [8, 17, 18]. Based on these, there is need to determine both the capsid and RdRp genotypes.

In most of Sub-Saharan Africa, routine norovirus surveillance is lacking or limited. Prevalence of norovirus in Africa among symptomatic children is estimated at 13.5% (95% CI 0.8–25.5) and among the asymptomatic at 9.7% (95% CI 7–31%) [10]. A previous study in Botswana has estimated norovirus prevalence at 24% [19] among symptomatic children. Following multiple gastroenteritis outbreaks that claimed many lives, the rotavirus vaccine was introduced in Botswana in July 2012 [20, 21]. While this move has significantly reduced cases and deaths related to gastroenteritis [22], understanding the causative agents of the remaining gastroenteritis cases is critical for prevention and management. Studies report that where rotavirus vaccine is used on a large scale, norovirus infections become significant [23]. It is therefore critical to understand norovirus infections in Botswana after a large scale-up of the rotavirus vaccine. In this study, we determined the prevalence, genetic diversity and trends of norovirus infections among children 5 years and younger admitted with acute gastroenteritis in Botswana between 2013 and 2015.

## Methods

### Study population and setting

Between July 2013 and December 2015, faecal samples were collected from children under 5 years admitted with acute gastroenteritis at four health facilities in Botswana (Princess Marina Hospital, Nyangabgwe Referral Hospital, Letsholathebe II Memorial Hospital and Bobonong Hospital), as part of the National Surveillance on Rotavirus Vaccine Impact Survey 2012–2015 [22]. The facilities represent geographical regions where more than 80% of Botswana’s population resides.

### Sample collection and preparation

Bulk stool (~ 5 ml) or flocked rectal swab was obtained from each participant within 48 h of hospitalization to avoid detection of hospital-acquired infections. All samples were transported to the National Health Laboratory on ice and kept at -80 °C until testing. A 10% stool suspension was prepared using Precellys® Lysing kit (Bertin Corp, Rockville, MD), according to the manufacturer’s instructions. The supernatant was then stored at -80 °C prior to nucleic acid extraction.

### RNA extraction and viral detection

Norovirus was detected using multiplex real-time PCR as described previously [24]. Briefly, total nucleic acid extraction was performed using easyMag (bioMerieux, Lyon, France). 200 μl of the supernatant was used to yield a nucleic acid elution volume of 70 μl. Norovirus GI/GII was detected using the viral multiplex panel and qualitative real-time PCR with the ABI 7500 Fast (Applied Biosystems, Carlsbad, CA, USA). The samples were taken through 45 PCR cycles. Targets with Cycle threshold (Ct) values below 40 were considered positive for that particular sample. For quality check, there were three controls included in every batch of testing in the assay: extraction negative control, in-house positive control, and master mix/reagent negative control. The extraction negative control was used to ensure adequate extraction process; the in-house positive control was used to ensure detection of all available targets in the assay; master mix/reagent negative control was used to ensure there was no contamination in master mix preparation.

### Capsid and RdRp gene amplification

Following norovirus detection, positive samples were tested by conventional RT-PCR using the Transcriptor One-Step RT PCR Kit (Roche Diagnostics, Mannheim, Germany). The capsid gene (5′ end, region C) was amplified with oligonucleotide primer sets G1SKF/R and G2SKF/R to amplify the 329 bp of GI and 342 bp GII capsid genes respectively, as previously described [25]. Each 50 μl reaction mixture contained 1 μl of each primer, 1 μl Taq DNA polymerase and 5 μl of cDNA. The PCR was performed under these conditions: 50 °C for 30 min, 94 °C for 10 min followed by 40 cycles, 94 °C for 30 s, 50 °C for 30 s, 72 °C for 1 min and final extension at 72 °C for 5 min. The RdRp gene was amplified using published oligonucleotide primer set, JV12/JV13 [26]. The reactions conditions were as follows: Each 50 μl reaction mixture, contained 2 μl each primer, 1 μl Taq polymerase and 15 μl of cDNA. PCR conditions were 50 °C for 30 min, 94 °C for 10 min followed by 40 cycles 95 °C for 30 s, 37 °C for 90 s, and 72 °C for 2 min and final extension of 5 min. The PCR products were visualised on agarose gel.

### Sequencing and phylogenetic analysis

The amplicons from the partial gene regions for the RdRp and capsid were purified using QIAquick PCR Purification Kit (Qiagen Inc., Valencia, CA), nucleotide sequencing was done using BigDye® Terminator v3.1 Cycle Sequencing Kit (Applied Biosystems, Carlsbad, CA) on 3130 DNA genetic analyser (Applied Biosystems, Carlsbad, CA). Sequencing was done in both directions using same specific primers used for RT-PCR. Sequences were edited using Sequencher® version 5.4.6 DNA sequence analysis software, Gene Codes Corporation, Ann Arbor, MI USA http://www.genecodes.comand norovirus strains were determined by nucleotide sequence analysis using the online Norovirus Genotyping Tool Version 1.0 [27] available at www.rivm.nl/mpf/**norovirus**/typing**tool** and confirmed using maximum likelihood method based on the Kimura 2-parameter model as implemented in Molecular Evolutionary Genetic Analysis (MEGA) software version 6.0 [28]. Robustness of the trees was assessed using bootstrap analysis of 1000 replicates. The sequences generated have been submitted to GenBank. Accession numbers for RdRp gene sequences are MF817977 to MF818009 and for capsid gene sequences the accessions numbers are MF920449.1 to MF920478.1. The GII.4 strains that were unidentified using the Norovirus Genotyping tool were identified using the online NCBI databases (https://blast.ncbi.nlm.nih.gov).

### Statistical analysis

Categorical variables and proportions were compared using a two-sided Chi-square or Fisher’s exact test where appropriate. Ninety-five confidence intervals (95% CI) were estimated using the binomial exact method and *P*-value of < 0.05 was considered statistically significant.

## Results

### Clinical and epidemiological data

Clinical and epidemiological data are given in Table 1. From July 2013 to December 2015 a total of 484 participants were enrolled in the study, 269 (55.6%) males and 215 (44.6%) females. The median age was 9 months (Q1, Q3: 6, 14; range 1 to 56 months). Participants were enrolled from the following hospitals as follows Princess Marina, 255 (53%); Nyangabgwe Referral, 112 (23%); Letsholathebe II Memorial, 83 (17%) and Bobonong, 32 (7%).

**Table 1:** Demographic and clinical characteristics of a study conducted in children with gastroenteritis in Botswana, 2013–2015

Characteristic	Total Tested	No. of norovirus Positive (%)	No. of norovirus negative (%)
Gender, *n* = 484			
Males	269	26 (9.7)	243 (90.3)
Females	215	19 (8.8)	196 (91.2)
Age (months), *n* = 484 (43 missing age)			
0 to 6	129	14 (10.9)	115 (89.1)
7 to 12	166	24 (14.5)	142 (85.5)
13 to 18	80	5 (6.2)	75 (93.8)
> 19	66	0	66 (100)
Facility, *n* = 484 (2 unknown facility)			
Princess Marina Hospital	255	28 (11)	227 (89)
Nyangabgwe Referral Hospital	112	11 (9.8)	101 (90.2)
Letsholathebe II Memorial Hospital	83	3 (3.6)	80 (96.4)
Bobonong Hospital	32	3 (9.4)	29 (90.6)
Year, *n* = 484			
2013	243	22 (9.1)	221 (90.9)
2014	141	10 (7.1)	131 (92.9)
2015	100	13 (13.0)	87 (87)
Symptoms, *n* = 484 (14 missing symptoms)			
Diarrhoea and vomiting	360	30 (8.3)	330 (91.7)
Diarrhoea only	62	6 (9.7)	56 (90.3)
Vomiting only	48	6 (12.5)	42 (87.5)

### Norovirus prevalence

The results for the norovirus prevalence are given in Table 1. Of the 484 participants, 45 were positive, 9.3% (95% CI 6.7–11.9). The prevalence for 2013, 2014 and 2015 were 9.1% (22/243), 7.1% (10/141) and 13.0% (13/100), respectively. The prevalence rates for 0–6 months, 7–12 months and 13 to 18 months were 10.9% (14/129), 14.5% (24/166) and 6.3% (5/80), respectively and these were not statistically different. There were no norovirus infections detected in the months of April, June and November for all the three years of the study. However, the infections were more likely to occur in the wet than in the dry months, (OR = 3.4; 95% CI 1.31–8.81). Diarrhoea was associated with vomiting symptoms, 360/470 (83%) (Fisher’s exact *p* = 0.001) and the majority of norovirus positive children presented with vomiting and diarrhoea, 30/45 (67%).

### Norovirus genotyping

**Fig. 1:**
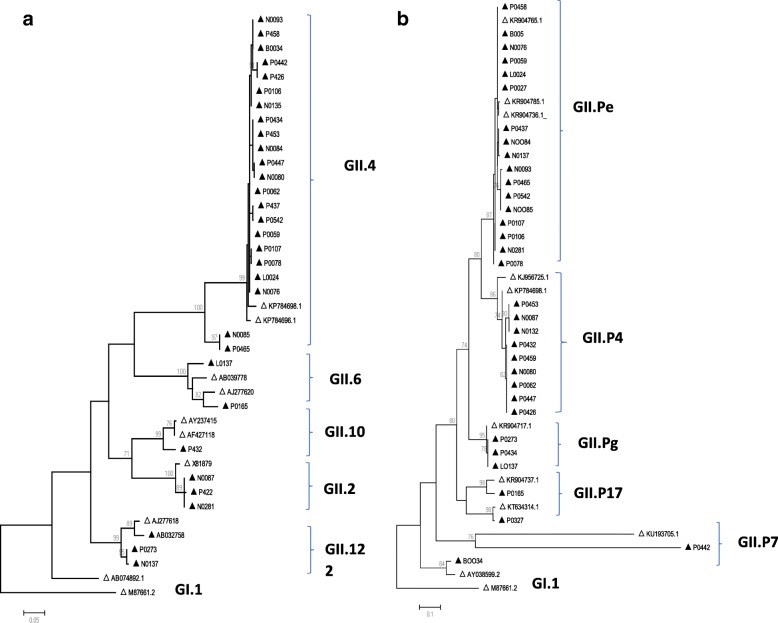
Phylogenetic tree of noroviruses based on (**a**) partial capsid and (**b**) RdRp genes of norovirus strains detected in children with acute gastroenteritis in Botswana, 2013–2015. The study samples are in shaded triangles and reference strains (unshaded triangles) are indicated by GenBank accession numbers. The scale bar represents nucleotide substitutions per site and the number above each branch corresponds to the bootstrap value. Scale bar is proportional to genetic distance

**Fig. 2:**
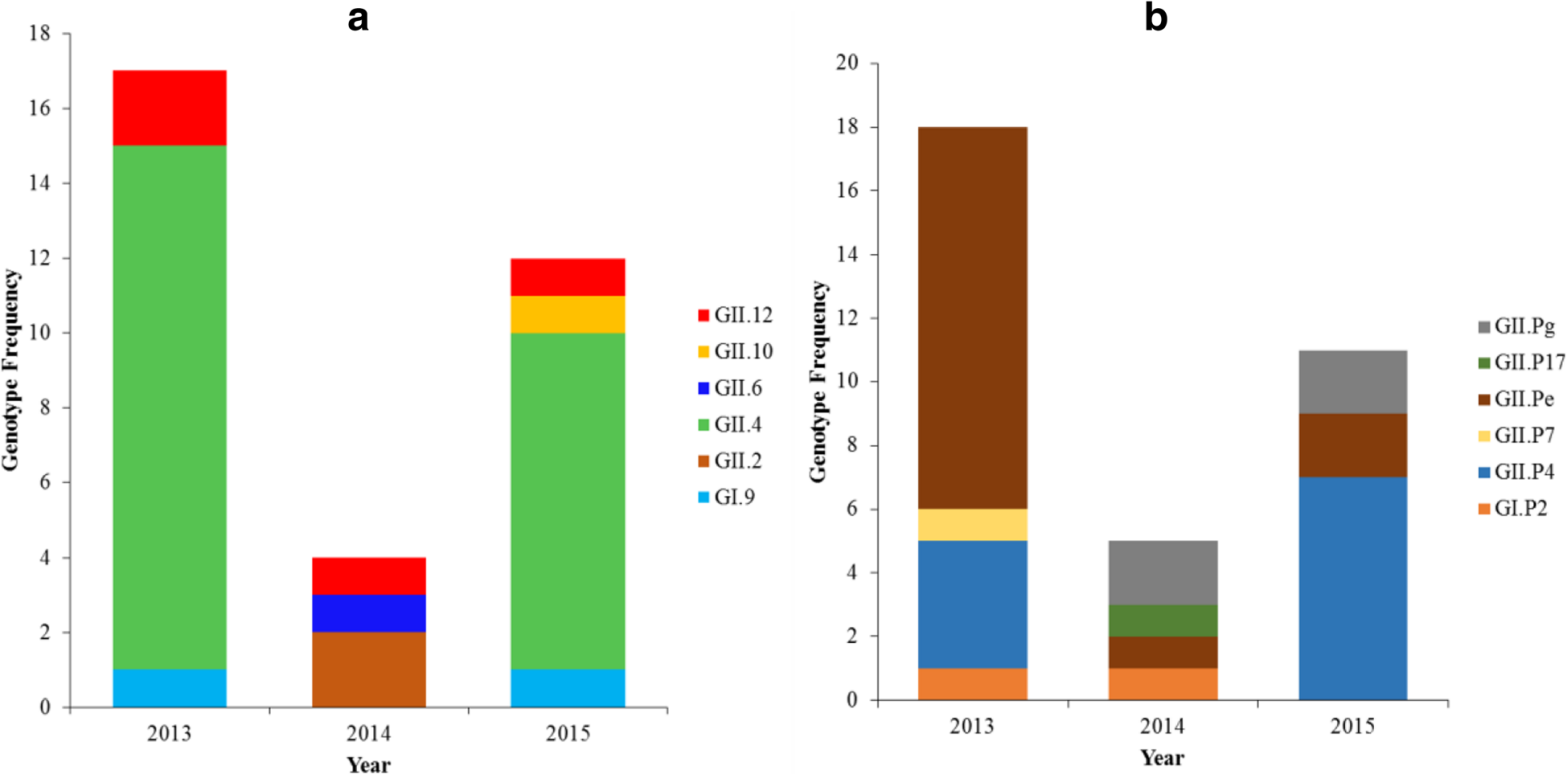
Distribution of norovirus genotypes by year from Botswana, 2013–2015. **a** Capsid and **b** RNA dependent RNA polymerase

From 45 positive norovirus samples, 33 (73.3%) were genotyped. Norovirus geno-grouping revealed that GII was most prevalent 93.9% (31/33) followed by GI at 6.1%, (2/33). In total 6 RdRp-based (33 samples) and 5 capsid-based (30 samples) genotypes were identified in GII geno-group as represented in Fig. 1a and b. Based on the capsid gene, the GII.4 strain was predominant with 69.7% (23/33) prevalence, other strains identified were GII.2 and GII.12 each at 9.1% (3/33); GI.9 strain at 6.1% (2/33); GII.6 and GII.10 strains each at (1/33), 3.0% (Fig. 1a). Regarding classification by the RdRp gene, GII.Pe was most prevalent at 50% (17/34), followed by GII.P4 at 29% (10/34). The other identified genotypes were GII.Pg at 9% (3/34), GII.P7 at 6% (2/34), GII.P2 and GII.P17 each at (1/34), 3% (Fig. 1b). As shown in Fig. 2a, in 2013 and 2015 based on capsid gene, the predominant strain was GII.4 but interestingly this strain was not detected in the year 2014. Norovirus infection in 2014 was characterized by GII.12, GII.6 and GII.2 strains, however based on the RdRp; GII.Pe was detected in combination with GII.2.

Of the 23 identified GII.4 strains, 13 were identified on the Norovirus Genotyping Tool as phylogenetically related to the Sydney 2012 variant, and 10 were unassigned. The unassigned samples were queried in the NCBI database (Additional file 1: Table S1) and 9 samples had close identity to the Sydney 2012 variant isolated in South Africa while one had close identity to a variant isolated in Japan. Five of these nine GII.4 strains were closely related to GII/Hu/ZA/2012/GII.Pe-GII.4 Sydney 2012/Johannesburg 9814 (KJ710247.1). Their query coverage ranged from 95 to 97% and nucleotide identity was 98–99%. The other 4 strains were closely related to the GII/Hu/ZAF/2013/ GII.Pe-GII.4 Sydney 2012/Cape Town (KR904238.1) with query coverage of 99–100% and 95–99% nucleotide identity. The last sample was similar to NV/Saitama T37Dgii/01/JP (AB112236.1) with 96% query coverage and 95% nucleotide identity, Additional file 1: Table S1. Based on the RdRp genotype sequences, in 2013 the GII.Pe strain was the most prevalent whereas in 2015, the GII.P4 was dominant, Fig. 2b. The GII.P4 strains were mainly New Orleans 2009 variants. Combined RdRp and capsid genotypes of 31 GII strains were determined resulting in 11 possible variants, of which 23/31 (67.7%) were concordant between the two classification and 10/31 (32.3%) were discrepant. These were GII.Pe/GII.4 at (36%, *n* = 11/31); GII.P4/GII.4 at (26%, *n* = 8/31); followed by GII.Pe/GII.12; GII.Pg/GII.4; and GII.Pe/GII.2 each at (7%, *n* = 2/31) and lastly; GII.P4/GII.10; GII.Pg/GII.12; GII.Pg/P2; GII.P2/GII.4; GII.P7/GII.4; GII.P2/GII.6 at (3%, n = 1/31) each, Table 2**.**

**Table 2:** Norovirus polymerase and capsid genotypes obtained from faeces of children with gastroenteritis in Botswana, 2013–2015

Polymerase (RdRp) genotype	Capsid (VP1) genotype	Number (%)
GII.Pe	GII.4	11 (36%)
GII.P4	GII.4	8 (26%)
GII.Pg	GII.4	2 (7%)
GII.Pe	GII.12	2 (7%)
GII.Pe	GII.2	2 (7%)
GII.P4	GII.10	1 (3%)
GII.Pg	GII.12	1 (3%)
GII.Pg	GII.2	1 (3%)
GII.P2	GII.4	1 (3%)
GII.P7	GII.4	1 (3%)
GII.P2	GII.6	1 (3%)

## Discussion

Viral gastroenteritis remains an important cause of morbidity and mortality in the developing countries. Furthermore, with the considerable decline of rotavirus-associated diarrhoea in countries that have introduced the rotavirus vaccine, it’s important to account for the high numbers of diarrhoea cases that are still seen. In this study, we report a possible shift in the variant distribution of norovirus infections in Botswana. The prevalence of norovirus was found to be at 9.3% among children admitted with acute gastroenteritis at four health facilities in Botswana between July 2013 and December 2015. These facilities are distributed across the southern and northern regions of the country where more than 80% of the population resides. The prevalence estimate falls close to the regional average of 13.5% (range 0.8 to 25.5) [10] but lower than that of the previous study in Botswana at 24% [19]. This may suggest a possible decline in the NoV infections in the last 10 years but not necessarily a reduction in the number of gastroenteritis cases. The prior study had a smaller sample size (collected over a 7 year period, ~ 10 samples per year) and did not only enrol children admitted to hospital. In the previous study, there was a high prevalence on norovirus and GII.4 and it is possible that this could have been due to outbreak of new GII.4 variant. There is over ten-year gap between sample collection of the study and our study. In this study, we have increased the sample size by prospectively enrolling each child admitted to hospital, determined the seasonality and included more geographic areas to understand the current burden of norovirus in the country. Additionally, it is possible that this represents a shift in the aetiology of diarrhoea and that the children could be exposed to wider causes of gastroenteritis. A study that characterized the aetiology of infections in a diarrhoea outbreak that occurred in 2006 in Botswana, revealed diverse aetiology of infections, mainly due to *Cryptosporidium parvum, Cryptosporidium homins* and *Escherichia coli*, while viral pathogens contributed a smaller proportion [29]. We also found the high number of gastroenteritis cases in spring but with low norovirus prevalence, which may be indicative of a different cause for the gastroenteritis.

While the prevalence is not statistically significant across the years, there is an upward trend that was observed between 2013 and 2015. Although causality cannot be ascertained at this point, it is possible that this significant increase could be attributable to the success of the rotavirus vaccine, which was introduced in 2012 [22]. Studies from other regions have shown that where rotavirus vaccines are used on a large scale, there is an increase in the proportion of norovirus cases [10, 30]. By 2014 rotavirus vaccine rollout or coverage in Botswana among the under 2 years was reported to be over 70%, a move that may have significantly reduced rotavirus related gastroenteritis [22]. The norovirus infection among the age categories was not statistically significant though there was a trend of higher prevalence in the age category of 7 to 12 months. This trend is consistent with some of the published studies, which showed that norovirus infections were more common in the 7 to 12 months age groups [16, 31, 32]. One explanation given is that this is a period where kids begin to interact more actively with their environment and can be easily exposed to norovirus [5, 32]. Furthermore, this is also a period when solid foods are introduced and children are often weaned from breastfeeding and in regions where clean water is not always available, there could be increased exposure to contaminated feeds [33]. Norovirus infections from this study were detected in most parts of the year with exception of April, June and November for all the three years of the study. Nonetheless, a higher proportion of infections occurred in the wet months than in the dry season. While studies from some regions show distinct patterns in norovirus seasonality [34, 35], results from African countries have not shown well-defined seasonal patterns. A study by Mans et al. in South Africa, which borders Botswana on the southern side, has shown a comparable norovirus seasonal pattern with infections occurring in the spring and early summer [16]. In some countries like Burkina Faso and Nigeria norovirus infections peaked in the dry season [31, 36], whereas in countries like Malawi and Morocco norovirus infections peaked in the wet seasons [37–39]. A study in Tunisia did not show any clear seasonal peaks [40]. Several factors have been suggested to contribute to the seasonality of norovirus infection and they include rainfall, population density and human behaviour [10], and as these are varied across African countries they could perhaps explain the discrepancy observed in the seasonality of norovirus infection. Additionally, there is a disparity on the prevalence of norovirus infections by health facility. A small proportion of norovirus cases were detected in Letsholathebe II Memorial Hospital and one explanation is that the health facilities are in geographically distinct areas with different livelihoods. While there is a lot of movement between the other three locations, Letsholathebe is situated in a village with less interaction than the other three.

Consistent with other RNA viruses, noroviruses are genetically diverse even within genogroups. The results from this study showed that GII was the most commonly detected strain. These findings are consistent with those of previous reports from Botswana and elsewhere [5, 19]. We also found a dominance of the GII.4 strains. While this study detected GII.4 variants Sydney 2012 based on capsid gene and New Orleans 2009 based on the RdRp gene, a previous study in Botswana reported more diverse GII.4 variants like Farmington Hills 2007, Hunter 2004, Yerseke 2006a and New Orleans 2009 [19]. A possible shift in GII.4 variant distribution could be attributed to the high mutation rate and evolution of norovirus strains leading to introduction of novel strains. The analysis of combined capsid and RdRp genes also showed diverse norovirus infections. GII.Pe/GII.4 Sydney 2012 was the most prevalent. These results suggest the possibility of recombination between open reading frames ORF1/ORF2 overlap region. We observed several GII.4 variants that were unassigned (10/23) by the Norovirus Genotyping tool, but were resolved by querying the NCBI database (Additional file 1: Table S1). Most of these strains showed between 95 and 100% identity with Sydney 2012 strains isolated in neighbouring South Africa [17]. The most common combined capsid and RdRp was the GII.Pe/GII.4 Sydney 2012, these two genotypes were detected in major outbreaks in Australia [41], and thereafter became the predominant species. Some of these strains have been described before [8, 41] and some are possible novel recombinants. In this study the combined genotypes were determined from separate RdRp and capsid amplicons, as such they could also represent coinfections and not recombinant strains. In 2014, GII.Pe was detected in combination with GII.2. To confirm recombinant strains the RdRp and capsid sequences will need to be determined from a single amplicon overlapping the ORF1/ORF2 junction [8, 41], a subject for further study.

## Conclusions

In conclusion, this study has shown that norovirus strains circulating in Botswana are diverse and that there is a trend toward increased proportion of norovirus positive cases in the three years following rotavirus vaccine introduction. These infections increased in the wet season. The GII.4 strain showed a possible shift in the variant distribution in the country.

## Additional file

Additional file 1: **Table S1.** GII.4 Variants and similarity to published sequences. Query data from NCBI database of previously unassigned samples. (DOCX 24 kb)

## Abbreviations

HRDC: Health Research and Development Council
MEGA: Molecular Evolutionary Genetic Analysis
NoV: Norovirus
ORF: Open Reading Frame
RdRp: RNA-dependant RNA polymerase
RT-PCR: Real-Time Polymerase Chain Reaction
