# Supplementary material for: Genetic and epidemiological analysis of norovirus from children with gastroenteritis in Botswana, 2013–2015
Source: BMC Infect Dis. 2018 May 30;18:246. doi: 10.1186/s12879-018-3157-y (PMC5975618; doi:10.1186/s12879-018-3157-y)
Supplement: Supplementary file 1 — Table S1. GII.4 Variants and similarity to published sequences. Query data from NCBI database of previously unassigned samples. (DOCX 24 kb) [file 12879_2018_3157_MOESM1_ESM.docx]

**Table S1**

GII.4 Variants and similarity to published sequences.

| **Sample** | **Query Coverage** | **Nucleotide identity** | **Variant(accession number)** |
| --- | --- | --- | --- |
| L0024 | 97% | 99% | GII/Hu/ZA/2012/GII.Pe-GII.4 Sydney 2012 Johannesburg9814 (KJ710247.1) |
| N0080 | 96% | 98% | GII/Hu/ZA/2012/GII.Pe-GII.4 Sydney 2012 Johannesburg9814 (KJ710247.1) |
| P0426 | 99% | 99% | GII/Hu/ZAF/2013/ GII.Pe-GII.4 Sydney 2012 Cape Town (KR904238.1) |
| N0085 | 96% | 95% | NV/Saitama T37Dgii/01/JP(AB112236.1) |
| P0542 | 97% | 98% | GII/Hu/ZA/2012/GII.Pe-GII.4 Sydney 2012 Johannesburg9814 (KJ710247.1) |
| P0453 | 100% | 99% | GII/Hu/ZAF/2013/ GII.Pe-GII.4 Sydney 2012 Cape Town (KR904238.1) |
| P00447 | 95% | 98% | GII/Hu/ZA/2012/GII.Pe-GII.4 Sydney 2012 Johannesburg9814 (KJ710247.1) |
| N0084 | 100% | 98% | GII/Hu/ZAF/2013/ GII.Pe-GII.4 Sydney 2012 Cape Town (KR904238.1) |
| P0434 | 99% | 95% | GII/Hu/ZAF/2013/ GII.Pe-GII.4 Sydney 2012 Cape Town (KR904238.1) |
| P0465 | 83% | 95% | GII/Hu/ZA/2012/GII.Pe-GII.4 Sydney 2012 Johannesburg9814 (KJ710247.1) |
